# Supplementary material for: Evolutionary advantages of turning points in human cooperative behaviour
Source: PLoS One. 2021 Feb 9;16(2):e0246278. doi: 10.1371/journal.pone.0246278 (PMC7872229; doi:10.1371/journal.pone.0246278)
Supplement: S1 Data — (PDF) [file pone.0246278.s001.pdf]

Data availability statement for the paper "Evolutionary advantages of turning points in human cooperative behaviour"

Daniele Vilone, John Realpe-Gómez, Giulia Andrighetto

This paper is a theoretical and analytical work, with simulation results presented to support our conclusions. No original experimental data have been utilized.
The algorithm of the simulations is described precisely in the paper in Subsection 2.3, for further information about it, or to check the code, please contact the corresponding author, Dr. Daniele Vilone: daniele.vilone@gmail.com.

The algorithm can be used by any author to replicate the study, properly citing the original work.
