# Supplementary material for: Evolutionary advantages of turning points in human cooperative behaviour
Source: PLoS One. 2021 Feb 9;16(2):e0246278. doi: 10.1371/journal.pone.0246278 (PMC7872229; doi:10.1371/journal.pone.0246278)
Supplement: Attachment — Submitted filename: VRA_letter2referees_PLoS.pdf [file pone.0246278.s002.pdf]

Dear Dr. Han,

Thank you very much for giving us the opportunity to revise our manuscript titled “Evolutionary advantages of turning points in human cooperative behaviour” to be considered for publication in *PLOS One*. We greatly appreciate the reviewers’ constructive comments on our previous manuscript. We have seriously considered each of these suggestions and used them to strengthen our work, which now we repute worth of publication.

Below we respond in detail to each of your own and the reviewers’ comments. When changes have been made to the manuscript we either insert a section of text from the paper and/or we provide the page numbers of the revised manuscript where the revisions can be found (we provide also a highlighted version of the text with changes in red). For ease of reading, the concerns are in italicized font and our responses are in plain font.

Reviewer #1:

1) In the Introduction and Discussion, it is stressed that systems are benefited from being close to criticality. I would recommend to explain a bit more how criticality is defined in this context. For example, being close to criticality means that the system is not finding a stable point? When the system is "too stable" it may favor maladaptive behaviors but it also can reach a good stable point that cannot be reached if it is "oscillating" easily. An explanation and/or discussion clarifying this kind of questions would improve the understanding of the claims.

We thank the reviewer for rising this point, which allow us to clarify a concept which we realize was not suitably explained. In fact, a phase transition can take place either between equilibria or between non-equilibrium states (*i.e.*, phase transitions in frozen configurations). In our case, a social system far from criticality can be too "conformist", that is, the individuals keep on behaving as the rest of population even when the actual behaviour is not convenient, or too "anarchical" where its members act independently from the others and without any benefit for the population as a whole.
We clarified this concept in the Introduction, (pages 1-2), with a new sentence explaining briefly the concept of criticality, and changing the passage where we describe the consequences of being far away from criticality, in both ways. In particular, we removed the misleading expression "too stable" and substituted it with "not responsive enough".

2) The current work is based in results from one experiment. I recommend to discuss its general applicability (ideally using data or results from other works).

The reviewer is right, we took into consideration that one experiment because we could detect the turning point there. It would be certainly important to find new and more general instances of criticality in human behaviour, in order to generalize our findings, but to this aim it is necessary to conceive new *ad hoc* experiments. We clarified this point in the final paragraph of the Conclusions (pages 11-12), which we have modified and enlarged.

3) Authors fixed the parameters q and r to the values of the experiment and leave the parameter p free in some of their analysis. Why not to do the same with the two first ones? A deeper explanation and/or discussion about the meaning of the parameter p and why it is the key parameter would be advisable.

We thank the reviewer also for this observation, indeed the importance of parameter p deserved to be more stressed. We focused on p because it is the parameter multiplying the number of K, so that it can magnify or reduce the drive to cooperate due to the presence of cooperating neighbours. For this reason it is much more meaningful to check the transition as a function of it instead of q or r.
We added this discussion in Sec. 2.2 (pages 3-4).

4) Is p influenced by the number of All-C and All-D players and then by the initial conditions?*

No. In fact, p^* is defined starting from the definitions in Subsection 3.1.1: it is the value of p for which the probability to cooperate when the neighbours are all cooperators becomes equal to 1. Of course, as shown in Figure 1, the shape of the transition depends on the distribution of the players of every kind (all-C, all-D, MCCs). We clarified this point making the definition of p* explicit already in Subsec. 3.1.1 (final paragraph) and at the beginning of Subsection 3.1.2 (page 6).

5) The simulation setup shows a few little differences from the experiment. For instance, the number of rounds. Do the authors think that any of these differences could influence p, q, and r?

The parameters q and r for our simulations are set based on the experimental results, and p is treated as a variable to check how the dynamics changes according to its value, but p, q and r remain all fixed during each simulation. Therefore, the few differences with the experimental setup do not have any effect on them.

6) The reader could benefit from a bit more elaborate explanation of the meaning of \xi.

We thank the reviewer for this remark, because \xi deserved actually a better explanation. This quantity is the correlation length, which measures the spatial memory of the system and it is commonly used in statistical physics to characterize transitions. We have added the explanation in Subsection 3,4 (page 8) with the new reference [26], just before Equation (4).

7) One conclusion that is extracted from this work (and the experiment) is that people cooperate more if more individuals around them did so (it is almost in the core of the definition of MCC). However it has been shown that in repeated collective games sometimes that is not the case. For example, Martinez-Vaquero et al (2020) show that individuals that cooperates only when not enough individuals did so in the previous round to reach a given threshold emerge for given conditions. Could this behavior been influenced by the type of game (weak prisoner's dilemma)? A bit of discussion in this line could be interesting.

The reviewer is right, these phenomena are often deeply influenced by the details of the game or, in general, the type of interaction among agents. We observed the turning point in the experiment [16], and it would be certainly interesting to find other ones in different human interactions, but this is a perspective for future works. These considerations have been drawn in the new paragraph added in the Conclusions (pages 11-12).

8) It would be really interesting if it is possible to include some discussion relating these results (relationship of criticality and cooperation) with other studies in other fields (e.g, sociology, history, sustainability science, etc.)

Up to now, the concept of criticality in social system has been applied to biology (see references [15], [16] and [17]), and no clear and irrefutable sign of criticality has been found in experiments with humans subjects or in observation of society in real life. Of course, the role of criticality in sociology, history and related fields is potentially very important being linked with cooperation, conformism, trust, etc., but it will be properly dealt with in future works, when more data will be collected. We have added these considerations in the new final paragraph in the Conclusions (pages 11-12).

Reviewer #2

We thank the reviewer #2 very much for his/her fully endorsement of our paper.

Reviewer #3

1. Authors propose a model of prisoner's dilemma game from [1] on 2D lattice and tune the parameters in the way to reproduce certain features observed in the actual experiment with human players reported in [16]. It might be Illustrative for a reader to see this parallel more clearly, for example, by showing some theoretical model curves fitted with experimental data from [16] (such as Fig.1). This work can be seen as an important theoretical re-examination of [16], but I found that obscure reading the paper.

We thank the reviewer for his/her remark, which allows us to highlight further the similarity between the experimental results and the theoretical ones. Indeed, also the time behaviour of the cooperation level for the non-evolutionary model near p^* (as in the experiment) is very similar to the empirical results. We add an inset in Fig. 1 (page 7) to illustrate this point.

2. In this context, authors should distance their work clearly from both references [1] and [16]. Value added by this paper above that of [1] and [16] should be unquestionable and clearly stated already in Introduction.

We have addressed to this point in the Introduction, with the new sentence in the second paragraph at page 2.

3. On the technical side, authors consider agents / players playing a game on 2D lattice. What happens if this is generalized to a lattice with more than two dimensions? This could actually facilitate observations of criticality in the system, enabling the authors to study its role more closely. To clarify, I am not asking for new numerical simulations, but for authors to at least discuss this point. Wouldn't be interesting to make a setting where closeness to criticality is most easily studied?

In this work, we aimed at reproducing experimental results, therefore, we utilized the same topology of the experiment in [16]. Of course, the reviewer is right in pointing out that in higher dimensions the observation of criticality is in general easier. Such a systematic theoretical analysis can be a promising future work. We added these considerations at the end of Sec. 3 (page 10).

4. What always bothers me in studies of this sort is the following. Criticality, traditionally studied in the context of phase transitions and statistical physics, describes a system between two phases, such as between liquid and solid, e.g. water and ice. It is not immediately obvious how this concept can be translated to social systems. Would a common sociologist agree on such interpretation of phases and the order parameter? This of course cannot be answered here, but authors should at least make a stronger

argument why do they expect that being near a critical point would be favorable to a social system (assuming we can perfectly well define what such a point means)? I think this is the core theoretical argument behind the whole paper.

In the case of our paper, the two phases are the one with no cooperation at all ($p > p^*$) and with a mixed configuration ($p < p^*$), with total cooperation at p^*. In other cases (see for instance reference [16]), we can have a "conformist" versus an "individualistic" phase. More in depth, we refer to reference [11] for the concept of criticality in social/evolutionary systems, where under very general conditions it is shown how near critical points a population benefits in terms of global payoff. We have added some considerations about this point in the Introduction, at the end of the second paragraph (page 2).

5. Literature review is quite comprehensive and complete, yet authors may find it of interest to add below two references:
- Guazzini, F. Stefanelli, E. Imbimbo, D. Vilone, F. Bagnoli, Z. Levnajić, Humans best judge how much to cooperate when facing hard problems in large groups, Scientific Reports 9, 5497, 2019.
- A. Guazzini, D. Vilone, C. Donati, A. Nardi, Z. Levnajić, Modeling crowdsourcing as collective problem solving, Scientific Reports 5, 16557, 2015.

We thank the reviewer for pointing out these useful references. We added them at the beginning of the Introduction (page 1), being the new references [4] and [5].
